# Addressing Diabetes Distress in Self-Management Programs: Results of a Randomized Feasibility Study

**DOI:** 10.13023/jah.0303.06

**Published:** 2021-07-25

**Authors:** Ranjita Misra, Samantha Shawley-Brzoska, Raihan Khan, Brenna O. Kirk, Sijin Wen, Usha Sambamoorthi

**Affiliations:** School of Public Health, West Virginia University; Social and Behavioral Sciences, School of Public Health, West Virginia University; College of Health and Behavioral Studies, James Madison University; School of Public Health, West Virginia University; School of Public Health, West Virginia University; Texas Center for Health Disparities, University of North Texas Health Sciences Center

**Keywords:** Appalachia, diabetes distress, self-management, randomized control trial, HbA1c, faith-based

## Abstract

**Background:**

West Virginia ranks 1st nationally in the prevalence of hypertension (HTN; 43.8%) and diabetes (16.2%). Patients with type 2 diabetes mellitus (T2DM) are distressed over physical and psychological burden of disease self-management.

**Methods:**

This study investigated the effectiveness of an intervention to reduce diabetes distress and outcomes [glycemic control, blood pressure (BP)] among T2DM adults with comorbid HTN. Participants were randomized to a 12-week diabetes and hypertension self-management program versus a 3-month wait-listed control group. Trained health coaches and experts implemented the lifestyle program in a faith-based setting using an adapted evidence-based curriculum. Twenty adults with T2DM and HTN (n=10 per group) completed baseline and 12-week assessments. Diabetes distress was measured by using a validated Diabetes Distress Survey (17-item Likert scale; four sub-scales of emotional burden, physician related burden, regimen related burden, and interpersonal distress). Baseline and post-intervention changes in diabetes distress were compared for both groups; reduction in distress in the intervention groups are depicted using waterfall plots. The mean age, HbA1c and BMI were 55 ± 9.6 years, 7.8 ± 2.24 and 36.4 ± 8.8, respectively. Diabetes distress (total; mean) was 1.84±0.71.

**Results:**

Participants reported higher diabetes distress related to emotional burden (2.1±0.94) and regimen-related distress (2.0 ± 0.74); physician-related distress was the lowest (1.18±0.64). In general, diabetes distress reduced among intervention participants and was especially significant among those with HbA1c ≤ 8% (r=0.28, p=0.4), and systolic/diastolic BP ≤140/80 mm Hg (r=0.045, P=0.18).

**Implications:**

Findings suggest that lifestyle self-management programs have the potential to reduce diabetes distress.

## INTRODUCTION

Diabetes, a highly prevalent chronic condition in the U.S., impacts 30.3 million Americans (13% adults).^1^ Disease management requires patients’ adherence to routine diabetes care activities and prescribed medication regimens to maintain good glycemic control known to prevent or delay the early onset of complications.^2^ The daily burden of disease management negatively affects patients’ emotional well-being,^3^ especially in rural and medically underserved populations in West Virginia (WV) due to poor access to care, multimorbidity and low socioeconomic status.^4^ West Virginia ranks 1st in the nation with 16.2% of adults who reported having diabetes^5^; 78% of adults with Type 2 diabetes mellitus (T2DM) have comorbid hypertension.^6^ The burden of managing their diabetes impacts the long-term psychological and emotional well-being of individuals with diabetes and related comorbidities.^7^

Diabetes distress is considered one of the most important psychosocial concerns among adults with diabetes. It was first proposed by a group of psychologists and psychiatrists (in 1995) to understand the emotional distress felt by T2DM individuals.^8^ Individuals experience diabetes distress or the emotional response due to the diagnosis, burdens and demands of complex disease management regimen, challenges of interacting with providers, and/or inadequate support or indifferent interpersonal relationships.^9^ However, diabetes distress is distinctly different from clinical depression,^10^,^11^ which is characterized by the presence of defined symptoms but fails to consider the emotional stress and worries related to diabetes. Diabetes distress is frequently observed in T2DM individuals,^12^ but is an important and underdeveloped area of research. It is associated with poor self-care, glycemic control, low self-efficacy, and health outcomes.^11^,^13^,^14^ One study found that a 10% reduction in diabetes distress reduced HbA1c by 0.25% among individuals with T2DM.^14^ Barriers to successful self-care and diabetes programs include access, distance, and transportation, particularly in rural (3rd in the nation) and medically underserved communities in the state.^15^

Faith-based behavioral interventions have shown promise, but most have been with minorities and African American communities.^16^ For example, 12-week lifestyle interventions conducted in a faith-based setting (churches) among African–Americans in Georgia and North Carolina reduced participants’ weight and fasting plasma glucose.^17^,^18^ Diabetes self-management programs in WV are either hospital-based or clinic-based and require referrals from physicians; few have been implemented in community-based settings or in Appalachia, and none have examined if self-management education can reduce diabetes distress.^19^ Cultural tailoring of diabetes programs with community buy-in are important factors for program success due to the Appalachian culture of distrust and social determinants of health found among adults with diabetes in rural communities. Faith-based organizations represent a potential partner in health behavior interventions for rural areas.^20^,^21^

The research team had an established partnership with the church’s pastor and the community advisory board to address health equities in the county. The church not only provided easy access, parking, and social acceptability for participants, but also was a free community-based facility that included a large fellowship hall for educational sessions and assessments. The fellowship hall was adjacent to a fully functional kitchen that was used for cooking demonstrations as well as a gym for group exercises. Hence, the Diabetes and Hypertension Self-Management Program (DHSMP) was conducted in a United Methodist church as it provided an easily accessible and socially acceptable setting for the program. West Virginia is also a predominantly non-Hispanic white (NHW) population with 93% NHWs.^22^ The DHSMP intervention used a participatory approach and a church advisory board to address cultural appropriateness of the multicomponent behavioral intervention, and health behaviors were adapted to the cultural context in Appalachia. A randomized controlled trial (RCT) design was used.

As part of a larger study to assess changes in HbA1c and blood pressure, this research compared changes in diabetes distress among 20 participants randomized to a 12-week DHSMP intervention (n=10) or a wait-listed control group (n=10) and received DHSMP after 12-week post intervention. The intervention was based on self-determination theory (SDT), and comprehensive assessments were conducted for both groups at 0, 3, and 6 months.

## METHODS

### The DHSMP Intervention

The DHSMP was a multicomponent, theory-based, randomized control, behavioral intervention trial designed to improve dietary intake, physical activity (PA), medication adherence, and coping with the disease for adults with comorbid T2DM and hypertension. Trained health coaches (HCs) and experts delivered the educational program and completed the assessments. Health coaches were students at West Virginia University that represented several majors (Public Health, Social Work, Nutrition, and Exercise Physiology). The health coaches were part of the implementation team and were provided two days of training by a multidisciplinary team of experts (comprised of public health behavioral interventionists, physician, registered dietitian, and pharmacist) on the evidence-based curriculum and session materials that included instruction and practice sessions, motivational interviewing, and standardized assessments. HCs were also trained to provide support for adherence to self-care activities and feedback during weekly communication with participants.

The direct observation method was used and implantation fidelity audits conducted to ensure that all HCs adhered to the same protocol for each educational session. A standardized audit form was completed for each HC during their mock presentations to study personnel. HCs received feedback to ensure components of the session are taught correctly and in the prescribed order as per protocol. Direct feedback was provided privately to the audited HCs for deviations from the protocol including any issue potentially affecting other HCs and the program staff. There was a 96% adherence to the standardized fidelity checklist for the current program.

The DHSMP curriculum was developed by utilizing three evidence-based, widely accepted, and scientifically acclaimed disease self-management programs. It incorporated lifestyle modification and education skills from the Centers for Disease Control and Prevention (CDC)’s National Diabetes Prevention Program (DPP).^23^ The second curriculum was from the Association of Diabetes Care and Education Specialists (ADCES7) self-care behaviors that provides 7 key behavioral areas for modifications and management for people with T2DM (healthy coping, healthy eating, being active, monitoring blood sugar levels, taking medication, problem solving and reducing risks).^24^ Lastly, guidelines from the Eighth Joint National Committee (JNC8) included self-management of hypertension.^25^ For example, the Dietary Approaches to Stop Hypertension or DASH eating plan that has shown to lower high blood pressure and improve cholesterol levels were incorporated into the healthy eating session. Physical activity, cooking demonstrations and other interactive activities were also included in the program sessions.

Briefly, the intervention included the following components:

1. 75-minute group educational session per week for 12 consecutive weeks (each 75-minute session included these components: weigh-in, group sharing and problem-solving regarding behavior change goals, and action plans from the previous week);
2. self-help educational materials for each session, including a CalorieKing book that provided macro and micronutrition information for individual and fast-food items and healthy eating recommendations;
3. a PA guide, educational demonstrations on how to use resistance bands, chair exercise/stretch and other general exercises and the intensity needed to achieve health benefits; pedometers were provided for daily use and tracking; and
4. weekly follow-up HC/participant communications (10–15 minutes) provided the opportunity to answer questions, provide continuous feedback on initiation and maintenance of health behaviors and reinforcement of health education messages.

Diabetes education programs often incorporate behavior-change theories. The goal-oriented facilitated group sessions used in the DHSMP were underpinned by the Self Determination Theory (SDT).^26^ According to SDT, patients are motivated to improve their health when they have the competencies and skills to self-manage their chronic conditions. Satisfaction to achieve the three basic human needs (autonomy i.e., to have control; competence for skills; and relatedness or desire to feel understood or cared by others) influence the initiation and maintenance of human behaviors. Goal setting during program sessions enhanced self-regulation and monitoring, knowledge through experiential learning, reinforcement, and support lead to outcomes. The SDT has been used in health behavior interventions and has yielded positive outcomes.^26^

Participants were recruited by utilizing electronic health record chart messages, hospital/university listservs, flyers, and community presentations. The eligibility criteria were based on individuals who were at least 18 years of age, BMI ≥25.0 kg/m^2^ and a reported diagnosis of diabetes and hypertension. Participants completed a brief screening to confirm eligibility and were provided a brochure describing the study. Informed consent was obtained prior to the baseline assessment. Participants were assigned to a HC and selected the mode of weekly communication (telephone/text/email) as per their preference. Food and activity logs were encouraged and collected from the participants at the weekly educational sessions. HCs also provided supportive feedback on participants’ weekly food and activity logs and encouraged autonomy and supportive interactions. Cooking demonstrations and PA skill-building exercises emphasized key concepts. All sessions were video-recorded, and participants who missed the session were provided a closed YouTube link. In addition, the program provided opportunities for sharing and learning in a group format to improve knowledge regarding diabetes, as suggested by the American Association of Diabetes Care and Education Specialists, American Diabetes Association, and Joint National Committee guidelines.

### Data Collection and Measures

Baseline and post-program data were collected from the participants. The program assessed demographics, diabetes distress, glycosylated hemoglobin, and blood pressure at baseline and 12-weeks. All assessments were completed at the faith-based setting in the morning (7am–10am). Participants who could not attend were provided a letter to do their blood tests at the Medical Lab (located 1 mile from the church). The study protocol was approved by the Institutional Review Board at West Virginia University and informed consent was received from each participant.

#### Diabetes Distress

One of the most frequently used measures of diabetes distress is the Diabetes Distress Scale (DDS).^27^ This 17-item self-report questionnaire is used to gauge physician-related distress as well as problems related to diabetes self-management, self-care, and metabolic outcomes.^28^ Response options ranged from 1 =not a problem to 6= a very serious problem. The DDS has 4 subscales: emotional burden, regimen-related, physician-related, and interpersonal distress. Higher scores indicated higher distress. Clinically meaningful cut-points have been established in adults with T2DM, with a mean score greater than 2.0 indicating moderate distress, and scores greater than or equal to 3.0 indicating high distress.^29^ The total DDS score had excellent internal reliability in this sample (Cronbach's alpha = 0.93).

#### Glycosylated Hemoglobin [HbA1c], Blood Pressure, and Cholesterol

Trained phlebotomists drew blood for HbA1c and lipids (total cholesterol and triglyceride) using established standards. HbA1c and lipids were analyzed by the University Medical Laboratory associated with WVU Medicine Hospital in Morgantown WV. Two blood pressure measurements were obtained by trained research staff with one-minute between measurements, using equipment and procedures that meet the recommendations for blood pressure measurement (e.g., sitting down, feet not crossed, arm on table). Average systolic and diastolic blood pressure measurements were calculated for each participant.

#### Demographic Variables

Demographics included age, gender, marital status, education, income, self-reported physical and mental health, and body mass index (BMI, calculated from height and weight measured at baseline and 12-week assessment).

## DATA ANALYSIS

Descriptive statistics and univariate analysis were computed for participant demographic characteristics, diabetes distress and its subscales, and clinical factors (HbA1c, total cholesterol, triglycerides, and systolic and diastolic blood pressure). Assumptions of normality and homogeneity of variance were checked using histograms, and Levene's test, respectively. None of the variables violated these assumptions. Hence, mean and standard deviations (SD) were calculated for continuous parametric variables and reported as mean ± SD. Percentages were calculated for the categorical variables. Participants were measured at two time points (baseline or pre-intervention and 12-week post-intervention) on diabetes distress and clinical factors. Line graphs were used to assess changes in diabetes distress from baseline to 12-weeks, for intervention group participants. Analysis of variance at post-intervention between the two groups provided program changes in the intervention group participants as compared to the measures in the wait-listed control group. Statistical inferences were based on a significance level of p (two-sided) ≤0.05. Data were analyzed using SPSS/PC software version 26.0.

### Sample Size and Power Analysis

The sample size and statistical power were based on HbA1c and blood pressure. For the comparison between before and after intervention, a total sample size of 18 achieves 95% power to detect a 0.6-point difference in HbA1c and 2.5 mm/Hg difference in blood pressure using a 2-sided paired t-test at a significance level of 0.05, assuming a standard deviation of HbA1c of 0.6 and blood pressure of 2.5 mmHg (based on a prior study). For comparison between the 12-week DHSMP and control group at 3-months post intervention, this sample size achieves 80% power to detect a 0.85-point difference in HbA1c and 3.5 mm/hg difference in blood pressure using a 2-sided two-sample t-test at a significance level of 0.05. Assuming a 10% attrition rate, a total sample size of 20 participants was required for this feasibility study.

## RESULTS

Participants included individuals with T2DM and comorbid hypertension (n= 20). Of these, 95% completed at least one outcome assessment, 90% completed the 3-month assessment. Overall, those who did not complete either baseline or 3-month assessments were similar to the completers but tended to be a little older. The baseline characteristics of the intervention and control group showed no significant differences (p>0.05).

### Demographics

The mean age and BMI were 55 ± 9.6 years and 36.4 ± 8.8 kg/m^2^, respectively. The majority of participants were females (70%), and by self-report, non-Hispanic whites (85%), were married or in a stable relationship (73%), had a college degree (51%), and family income less than $50,000 (65%). Six percent of participants were of African Americans and Asian race, respectively. Approximately, half (48.5%) perceived their physical health to be fair/poor but reported their mental health as good to excellent (78.5%).

### Diabetes Distress

Mean baseline diabetes distress was 1.84 ± 0.71. Participants reported higher diabetes distress related to emotional burden (2.1 ± 0.94) and regimen-related distress (2.0 ± 0.74); physician-related distress was the lowest (1.18 ± 0.64). Using the scale scoring to convert diabetes distress scores to no or low (≤ 2) versus moderate/high distress (>2), overall, 20% of participants reported moderate to high diabetes distress. The highest level of distress was emotional burden (25%) of every day lived experience followed by regimen-related (20%) and interpersonal distress (20%), and the lowest was for physician-related distress (10%).

### Clinical Measures

Average HbA1c for the total sample at baseline was slightly over the recommend level 7.66 ± 1.50 (Intervention group 7.59 ± 1.37 versus control group 7.78 ± 1.77; p=0.76). Furthermore, baseline mean systolic blood pressure was 136.62 ± 17.98 mg/dl (intervention group 132.75±19.42 versus control group 132.80 ± 14.14; p=0.77) and mean diastolic blood pressure was 81.65±10.74 mg/dl (intervention group 80.44 ± 11.36 versus control group 83.60 ± 9.91; p=0.47). Participants reported taking multiple medications and averaged 1.3 BP and 1.4 glucose lowering medications, respectively. In general, almost one-third (29%) of participants exceeded the recommended HbA1c range of 7%–8% for glycemic control (18% had ≥9.0) and 30.3% had systolic and diastolic blood pressure over 140/90 mmHg.

**Figure 1:**
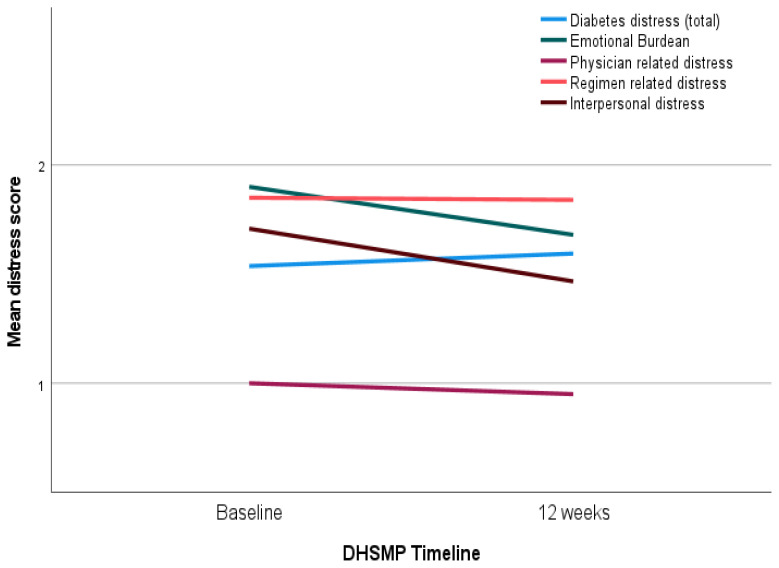
Pre and Post DHSMP Intervention Changes in Diabetes Distress and Its Four Domains in Program Participants DHSMP is a 12-week multi-component diabetes and hypertension self-management program. Mean (Y-axis) indicates changes in baseline and post-intervention (12-week) diabetes distress scores in the intervention group participants (n=10). Diabetes distress included total and 4 domains (regimen-related, emotional, interpersonal and physician related distress), calculated using the Diabetes Distress Scale (DDS) which had 17 Likert scale questions; response options were 1 (not a problem) to 6 (a very serious problem).

Table 1 presents baseline values and participants’ changes in diabetes distress and clinical measures (HbA1c, blood pressure and lipids) at 12-weeks post intervention (presented as mean [95% CI]). Significant differences were noted in reduction of interpersonal distress in the intervention group while it increased for the control group participants at post-test (p=0.02). Although not statistically significant, program participation lowered total diabetes distress and three other subscales among intervention participants as compared to the control group. Average distress of <2 shows no distress and ≥2 indicates moderate level. Figure 1 indicates baseline-post-program changes in diabetes distress and its four domains using line graphs.

Program changes in clinical measures of HbA1c, lipids (total cholesterol, triglycerides), blood pressure and BMI also showed a pattern of decrease in the post-intervention measurements (p>0.05). While not shown in the table, at baseline 40% of intervention group and 30% of control group participants had HbA1c ≥8.0. After 12-weeks of the DHSMP, 33% of the intervention group participants had HbA1c ≥8 while there was no percent change noted in control group participants (i.e., 30%). Similarly, at baseline, 37.5% of intervention group and 34.6% of control group participants had BP over 140/90 mmHg and 30% of intervention versus 34% of control group participants had abnormal BP levels at the 12-week assessment. This indicated 7% and 7.5% of intervention group participants lowered their lower their HbA1c and BP to an acceptable range after 12-weeks of program participation. These percent changes in adverse levels are considered to be clinically significant despite the mean changes not showing a statistically significant difference due to the small sample size. In addition, total diabetes distress reduced among participants with HbA1c ≤8% (r=0.28, p=0.04), and systolic/diastolic blood pressure ≤140/80 mm Hg (r=0.45, p=0.01; not shown in the table).

## DISCUSSION and IMPLICATIONS

Findings of this pilot feasibility trial demonstrate that a multicomponent behavioral intervention has the potential to reduce diabetes distress among a predominantly Non-Hispanic white rural population. More specifically, participation in the 12-week DHSMP reduced two types of diabetes distress among the intervention participants: emotional burden and regimen-related distress. One of the plausible explanations of reduction in the emotional burden (i.e., stress of managing diabetes, other life stressors) is the social support provided by the trained study personnel as well as from other participants during the group educational setting. Social support has been shown to reduce individuals’ emotional distress in a prior study.^30^ Among rural Appalachian individuals with diabetes, emotional burden caused the highest level of diabetes distress in the participants and plays a critical role in living and managing the disease.^27^,^31^ Moderate to high levels of emotional distress is associated with poor self-care behaviors such as poorer physical activity and dietary intake among patients and glycemic control.^32^ Regimen-related distress also declined after the intervention and concurs with a prior study that showed individuals have higher levels of regimen-related diabetes distress, predictive of HbA1c levels.^33^

Prior research has consistently shown that diabetes self-management education and support (DSME/S) is an integral part of diabetes care and improves glycemic status and health outcomes.^34^ The reduced levels of distress can be impacted by various factors such as an increase in knowledge and self-efficacy for physical activity, making heathier dietary choices, coping with life stress as well as motivation and feedback by their health coaches for pragmatic and incremental improvements for dietary modifications and physical activity. Perceived competence and motivation are constructs used by the SDT known to impact healthy behaviors.^35^

This study adds to the body of knowledge as few have focused on effectiveness of diabetes interventions to reduce diabetes distress in West Virginia adults with diabetes. These individuals have significantly more challenges of living with diabetes than their urban peers due to low socioeconomic status, access to health care, safe walking areas for physical activity and healthy food, lack of transportation, and financial worries.^36^ Hence, self-management programs, such as the DHSMP, provided useful knowledge and skill building exercises (e.g., cooking demonstrations as well as promoting walks and chair exercises for individuals with arthritis and back problems). It should be noted that the prevalence of multiple chronic conditions in WV, a state considered mostly rural and medically underserved, is higher than the national average; Hence, diabetes distress and self-care among T2DM individuals with co-occurring chronic conditions may be even more complex and challenging.^37^ A focus group study among adults with type 2 diabetes living in rural West Virginia described living life as an evolving process, being on guard as a vigilant ongoing responsibility, and awareness of changes to their body when facing life stress, potential problems and taking charge.^31^

Findings have implications for healthcare providers as they can identify and suggest lifestyle changes and coping strategies for diabetes distress in their patients. Routine screening for diabetes distress, as part of routine clinic evaluation, should be explored and coincide with the current recommendations for education and healthy lifestyle modifications to prevent and treat diabetes for prevention of complications and better quality of life.^15^,^38^ It can be a useful tool to identify individuals with higher distress for referrals to DSME/S. More specifically, educational programs should focus on distress related to patients’ regimen-related distress as well as interpersonal distress as they escalate the distress levels of individuals with T2DM.^39^ Provider understanding of the benefits of self-management programs can help build referrals as a critical component of the treatment plan. While most available self-management education/programs in WV are in clinic or hospital settings, referrals to community-based programs at no cost to the individuals, e.g., Dining with Diabetes or others similar to DHSMP that are available at local health departments or through community organizations can reduce diabetes distress in T2DM individuals while promoting healthy lifestyle modifications. In addition, organizations that deliver diabetes education programs may also consider marketing the positive experiences of patients to reduce diabetes distress and improve self-management of chronic conditions.

Research shows that diabetes distress impacts the daily lives of adults with T2DM and can lead to anxiety, depression, and effects on quality of life.^40^ In West Virginia, the geographical and social isolation can further contribute to increased distress levels as related to diabetes care and related services resulting in poor glycemic control.^2^,^3^ However, the results indicated that addressing diabetes distress in self-management programs can enable individuals to make informed choices critical for adherence to self-management activities (e.g., diet, physical activity, medication adherence).^4^ Furthermore, higher levels of emotional burden, interpersonal- and regimen-related distress among participants could play a significant role in glycemic control. Understanding of the patient’s comorbid chronic conditions and related distress is important since social support and provider interactions are severely limited in WV. According to SDT, these extrinsic factors can lead to lower self-determination and affect the behavior of individuals with T2DM.^35^ However, knowledge/education, positive feedback, and encouragement can boost patient’s self-determination by competence, connection, and autonomy ultimately leading to improved physical and mental well-being.

Although this study highlighted an important gap in the literature, there are some limitations to address. First, the sample size limited the power of the statistical analyses and generalizability. Second, there were a higher number of females who participated in the intervention program. Third, the small sample size did not allow control or assessment of individuals who attend church regularly, especially women, who tend to have better health outcomes than non-church goers. Additionally, studies have reported individuals with lower educational levels have higher diabetes distress. Thus, the effect of education level and religious support on diabetes distress in rural adults requires further evaluation. Lastly, findings on diabetes distress were based on self-reported measures included in the survey. However, this study used a rigorous study design to assess diabetes distress in West Virginians with diabetes. Future research should include a larger sample size, a more representative sample, and appropriate clinical metrics to examine diabetes stress.

## CONCLUSION

These findings showed that the intervention resulted in a decreased trend in diabetes distress (especially emotional distress and regimen-related distress) after 12 weeks among rural adults with diabetes. Routine clinic assessment for diabetes distress should be explored and coincide with the current recommendations for diabetes education and healthy lifestyle modifications.

**Summary Box**

**What is already known on this topic?** Comorbid diabetes and hypertension are dual epidemics in WV, an entirely Appalachian state, that has the highest prevalence of both chronic conditions in the nation. Diabetes distress is one of the most important psychosocial concerns among adults with diabetes.

**What is added by this report?** This research addresses community-based programs to reduce diabetes distress for rural adults. Twenty adults with comorbid diabetes and hypertension participated in a randomized control trial (12-week diabetes and hypertension self-management program) versus a 3-month wait-listed control group. Changes in diabetes distress were measured by using a validated Diabetes Distress Survey at baseline and post-intervention for both groups. In general, participants reported higher diabetes distress related to emotional burden and regimen-related distress. Findings showed the intervention resulted in a decreased trend in diabetes distress, especially among those with well-controlled blood sugar and blood pressure.

**What are the implications for future research?** Further research is needed to illustrate program effectiveness in reducing diabetes distress in larger sample. In addition, the impact of educational level and attendance of church services on diabetes distress should be explored. This work provides support for interventions to address mental health and well-being for adults with comorbid chronic conditions.

## Figures and Tables

**Table 1:** Baseline and Post-program Changes in Diabetes Distress and Clinical Factors in DHSMP Intervention vs Control Group Participants

Program Variables	Intervention Group (N=10)		Control Group (N=10)		Program Change
	Baseline Mean (95% CI)	Post program Mean (95% CI)	Baseline Mean (95% CI)	Post (12-week) Mean (95% CI)	F-value (p value)*
**Diabetes Distress**					
Total DDS score	1.65 (1.1, 2.1)	1.76 (0.9, 2.5)	1.59 (1.1, 2.1)	2.09 (1.5, 2.7)	2.70 (0.12)
Regimen-related distress	1.92 (1.3, 2.7)	1.85 (1.0, 2.7)	2.02 (1.2, 2.8)	2.18 (1.7, 2.7)	1.03 (0.32)
Emotional distress	1.95 (1.1, 2.4)	1.68 (1.4, 2.0)	1.94 (1.5, 2.3)	2.30 (1.4, 3.2)	0.72 (0.41)
Interpersonal distress	1.70 (1.0, 2.4)	1.60 (0.9, 2.2)	1.46 (0.9, 2.0)	2.50 (1.7, 3.3)	6.23 (0.02)
Physician-related distress	1.25 (0.6, 1.3)	0.97 (0.6, 1.3)	1.00 (0.7, 1.8)	1.22 (0.9, 2.0)	2.96 (0.10)
**Clinical Factors**					
Triglycerides	179.80 (137.5, 222.1)	169.78 (91.3, 248.2)	143.78 (80.0, 207.5)	188.10 (109.3, 266.9)	0.14 (0.71)
Total Cholesterol	155.13 (137.2, 173.1)	156.11 (132.0, 180.3)	176.67 (137.9, 215.4)	190.40 (153.0, 227.8)	2.91 (0.10)
HbA1c	7.59 (6.8, 8.4)	7.20 (6.2, 8.2)	7.79 (6.4, 9.2)	7.77 (5.9, 9.6)	0.35 (0.55)
BMI	36.55 (32.4, 40.7)	34.07 (28.2, 39.9)	35.61 (30.1, 41.2)	34.83 (29.5, 40.1)	0.05 (0.82)
Systolic BP	132.75 (122.4, 143.1)	128.70 (120.8, 136.6)	142.80 (132.7, 152.9)	139.60 (125.9, 153.3)	2.43 (0.13)
Diastolic BP	80.44 (74.4, 86.5)	81.58 (77.0, 89.8)	83.60 (76.5, 90.7)	85.80 (67.0, 104.6)	0.08 (0.78)

* Program change was calculated by the difference in post-intervention measures between the two groups; CI = confidence interval.

DDS = Diabetes distress scale score among intervention and wait-listed control group at baseline and end of the program (12-weeks). DDS subscales include regimen-related, emotional, interpersonal and physician-related distress.

HbA1c = glycosylated hemoglobin level; BP = blood pressure.

DHSMP is a 12-week multi-component diabetes and hypertension self-management program. Post-program assessments were done at 12-weeks
